# A Mechanistic Study on the Photocatalytic Conversion of Methane to Ethane on TiO_2_ and Au–TiO_2_ Nano Clusters

**DOI:** 10.1021/acs.jpca.5c03782

**Published:** 2025-09-26

**Authors:** Vidya Kaipanchery, Dorota Rutkowska-Zbik

**Affiliations:** 132074 Jerzy Haber Institute of Catalysis and Surface Chemistry PAS, ul. Niezapominajek 8, 30-239 Kraków, Poland

## Abstract

We have studied the mechanistic pathway of methane to ethane conversion on the TiO_2_ and Au–TiO_2_ nano clusters using Density Functional Theory calculation. The calculated reaction energies and energy of activation on the mechanistic pathways involved in the ethane formation reaction confirm the successful conversion of methane to ethane on the TiO_2_ nano cluster under normal conditions. The introduction of a gold nano cluster as a cocatalyst with the TiO_2_ nano cluster reduces the energy needed for the C–C coupling step in the ethane formation reaction. In the C–H activation reaction on the Au_6_–TiO_2_ nano cluster, the reaction energy is almost the same as that on the bare TiO_2_ nano cluster. The carbanion generated during the transition state drives the reaction forward in C–H bond activation and C–C coupling reactions. The final desorption energy of free ethane from the adsorbed surface is smaller in the case of the Au_6_–TiO_2_ nano cluster compared to the TiO_2_ nano cluster.

## Graphical abstract

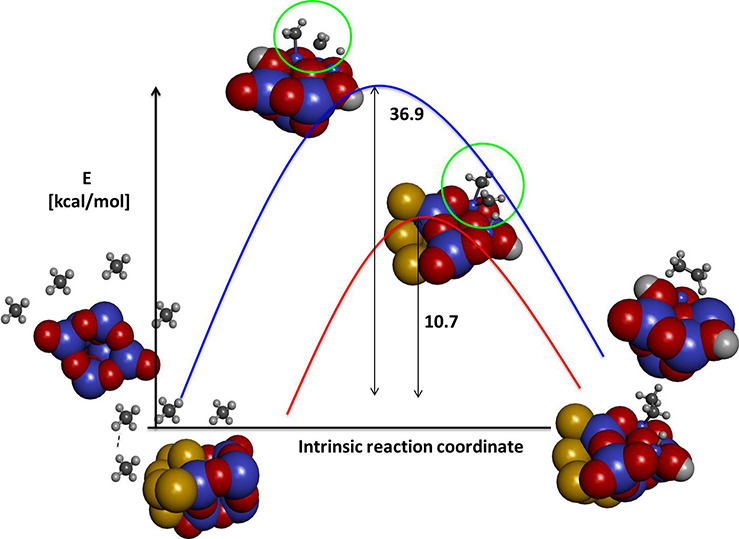

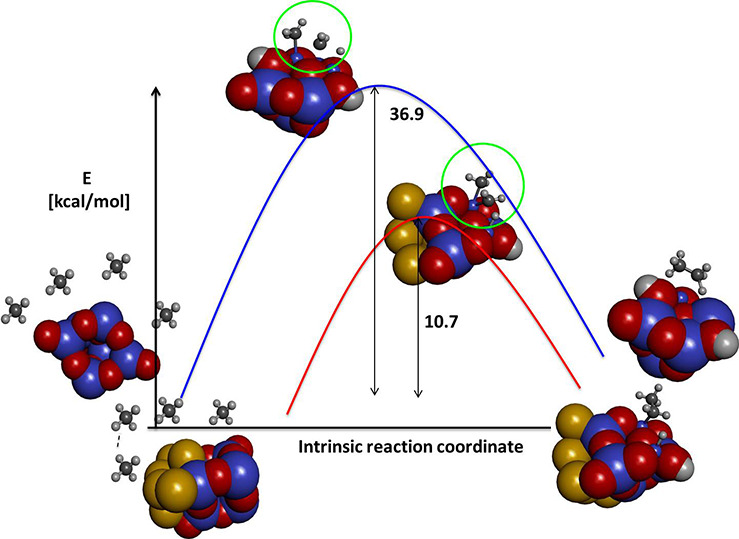

## 1 Introduction

Methane (CH_4_), the main constituent of natural gas and byproduct of oil recovery, is a major contributor to global warming, almost 25 times stronger than that of CO_2_.^1,2^ There has been increased drive on research for the direct conversion of CH_4_ to value-added hydrocarbons with high industrial potential like methanol (CH_3_OH), ethanol (CH_3_CH_2_OH), ethane (C_2_H_6_), ethylene (C_2_H_4_), etc.^3^ It is important to design green reactions that provide sustainable energy, a cleaner environment, and lesser emission of greenhouse gases in the coming future,^4^ as the global reliance on nonrenewable sources of energy has contributed to the growing environmental and climate challenges.^5^ Solar-driven energy generation using photocatalytic processes represents a sustainable solution to these challenges.^6–10^ Photocatalytic coupling of methane to ethane by using solar energy at room temperature is a promising approach for conversion of methane (CH_4_) to ethane (C_2_H_6_).^3,11–13^

The thermocatalytic degradation of the sp^3^-hybridized highly symmetric CH_4_ molecule with strong C–H bonds (434 kJ mol^–1^) requires a very high temperature and also results in low selectivity and CO_2_ emission. Therefore, alternative approaches, such as photo- or electrocatalysis, are in demand. The primary challenge in photocatalysis is to design catalysts that can efficiently utilize visible light in the solar spectrum, increase the yield of the desired product (here: ethane), and reduce undesired products like CO_2_. There has been intense interest in finding a green reaction for C–H activation of CH_4_. The nonoxidative coupling of methane (NOCM) to higher hydrocarbons is one way for breaking the C–H bond in CH_4_.^14^ The transition metal nano clusters (Ti, W, Mo, Fe, Ni, *etc.*) act as robust catalysts of the activation of C–H bonds.^15–19^

Typically, metal nano clusters are of nanometric size particles, composed of atoms of a single metal element (monometal) or multiple elements (alloys). The nano clusters adopt special atomic arrangements to reduce their surface energy, and their electronic structures become discretized compared to the continuous electronic structure in bulk metals. This discretization of the electronic levels in the metal clusters results in striking properties and characteristics. For the above-mentioned reasons, the metal nano clusters have attracted much attention as new functional nanomaterial and promising candidates for various applications in science and technology like catalysis, photoluminescence, biomedicine, and magnetism.^20–23^ In photocatalysis, the nano clusters are found to be promising compared to their bulk counterparts.^24–27^ Fujishima and Honda discovered that water splitting could occur over titanium dioxide (TiO_2_) under UV light irradiation, which sparked intense research in the fields of photocatalysis, photovoltaics, and sensors.^28^ TiO_2_ has a band gap of 3.0–3.2 eV, which limits its ability to absorb light only in the UV region of the spectrum.

It is essential to design catalysts where a cocatalyst added to a TiO_2_ nano cluster can extend its ability to absorb light also from the visible range and to improve the selectivity and efficiency of the photocatalytic reaction. The most recent illustration is the use of noble metals (Au, Ag, Pt, etc.) as cocatalysts, because their energy gaps for absorption are in the sunlight spectrum comprising near IR, visible, and UV wavelengths.^29–31^ Depending on the atomic species, size, shape, and chemical environment, the noble metal nano clusters can activate the photochemical reactions at the corresponding energy gaps. For potential catalysis application, it is important to produce very small clusters, as they usually appear to be more active than their bulk counterparts. The gold nano clusters have the ability to extend the spectral response in the visible region of TiO_2_.^32,33^ The Au nano clusters showed sensitization of TiO_2_, which created a large number of surface states. Thus, metal–semiconductor nano cluster Au/TiO_2_ catalysts are a promising way for ethane generation via green energy pathway.^15,24^

The current study involves methane to ethane conversion via a solar-energy-driven process on a TiO_2_ nano cluster as the photocatalyst. Our aim was to study the reaction mechanism on bare TiO_2_ as well as on the composite Au/TiO_2_ cluster to examine the influence of the cocatalyst on the reaction pathway.

## 2 Methods

Density functional theory (DFT) calculations using the Gaussian 09 suite of programs were carried out to study the mechanism of methane to ethane conversion on the TiO_2_ nano cluster.^34^ The TiO_2_ nano cluster adopted in this study is depicted in Figure 1. The oxygen vacancy at one of three possible sites (O1, O2, O3) is taken into account as the factor facilitating methane absorption, as indicated by other studies.^35^ Furthermore, the overall positive charge of +1 is also considered on the TiO_2_ cluster to represent in the DFT calculation the hole generated under photoexcitation, resulting in the TiO_2_^+^ nanocluster. Geometry optimization of all the structures studied is performed using the hybrid density functional B3LYP with all atoms and the LANL2DZ basis set with zero point correction in the gas phase implemented in Gaussian 09 program suite.^34^ The intrinsic reaction coordinate (IRC) calculations are done at the UB3LYP/LANL2DZ level for all the different reaction steps involved in methane to ethane conversion. The transition state is optimized using the QST3 method followed by the IRC path calculation.^36^ The energy of activation (*E*_act_) is calculated from the difference in energy of the transition state and reactant, and the reaction energy (Δ*E*) is calculated as the difference in energy of the product and reactant. The NBO population analysis is also done for the transition states in the different catalytic pathways.^37^

**Figure 1:**
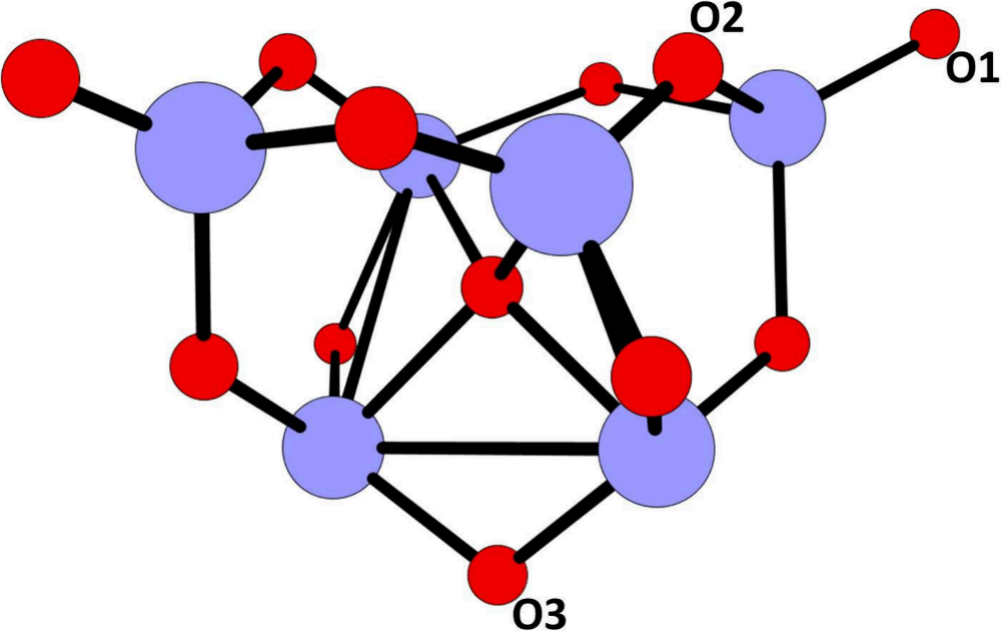
TiO_2_ cluster with three positions for oxygen removal marked as O1, O2, and O3 (Color coding: blue–titanium, red–oxygen, gray–carbon).

## 3 Results and Discussion

### 3.1 Methane to Ethane Conversion on a TiO_2_ Nano Cluster

We have studied the mechanism of adsorption of CH_4_ on a TiO_2_ nano cluster surface and its coupling to form ethane. The reaction steps involve: (1) adsorption of CH_4_ on TiO_2_, (2) C–H bond activation in the adsorbed CH_4_, (3) coupling of two CH_3_ species attached on the adjacent Ti atoms on TiO_2_, (4) ethane formation, and (5) desorption of ethane from TiO_2_.

#### 3.1.1 The Adsorption of CH_4_ on a TiO_2_ Nano Cluster

The catalytic conversion of CH_4_ to ethane was studied on a TiO_2_ nano cluster. We used a TiO_2_ nano cluster (Figure 1) as our substrate for adsorption of the CH_4_ molecule. The HOMO–LUMO gap in the TiO_2_ nano cluster is calculated to be 2.63 eV (∼470 nm in wavelength). The TiO_2_ nano cluster absorption gap energy falls in the visible range of the solar spectrum, thus enabling photochemical activity.

Three unique sites (vacancies in the O1, O2, and O3 positions, Figure 1) for methane binding on a titania nanocluster are studied within two scenarios—for an uncharged TiO_2_ nanocluster and for TiO_2_^+^ mimicking the photoexcited system—see Table 1 for the CH_4_ binding energies. The comparison of the CH_4_···Ti binding energies indicates that the hole creation on the metal nanocluster enhances methane binding on the cluster, and the oxygen removed from position O3 offers the most favorable methane binding site compared to other two positions, Figure 2 for the geometry structures of the resulting **1**, **2**, and **3** clusters in which CH_4_ stays in these positions, respectively. The interaction between CH_4_ and the TiO_2_ nano cluster atoms is classified as a nonbonding interaction. In the following, we considered the reaction mechanism proceeding on TiO_2_^+^, starting from the **3** structure.

**Table 1:** Binding Energy (in kJ/mol) for CH_4_ with Three Different Ti Atoms on a TiO_2_ Nano Cluster Calculated at the UB3LYP/LANL2DZ Level of Theory

structure	Binding energy with single oxygen vacancy (kJ/mol)	Binding energy with single oxygen vacancy and positive charge (kJ/mol)
**1**	–1.88	–3.56
**2**	103.85	31.46
**3**	–39.20	–67.03

**Figure 2:**
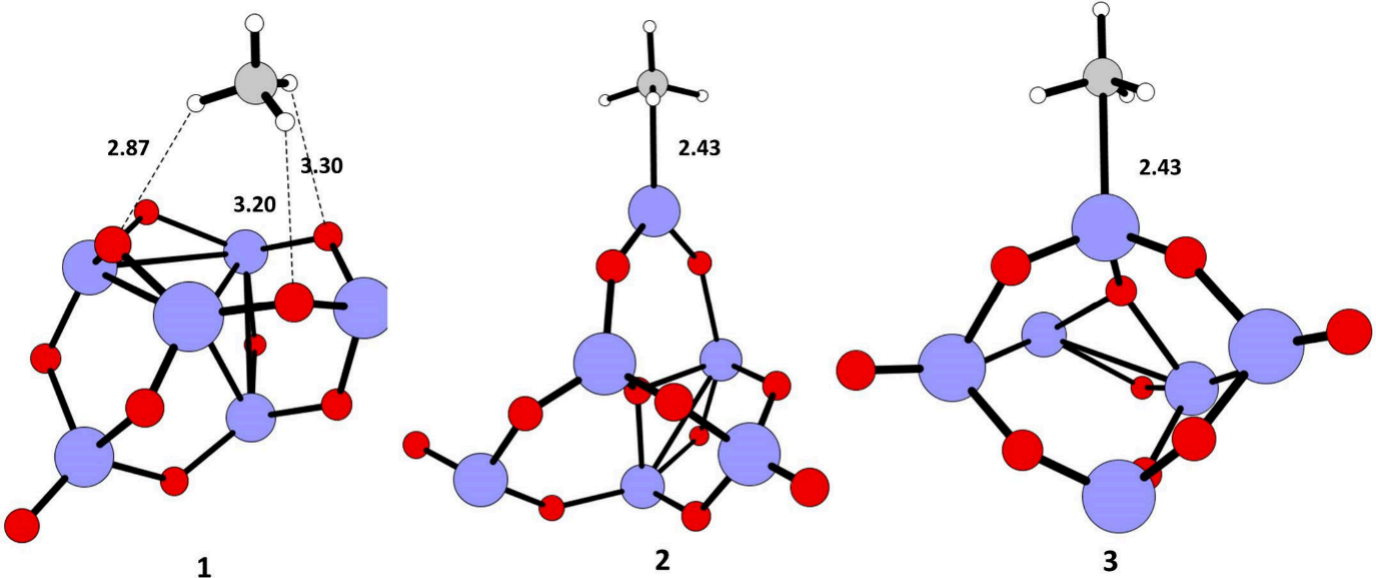
Optimized structures of TiO_2_–CH_4_ interaction with oxygen vacancy at different sites of TiO_2_ nano cluster calculated at the UB3LYP/LANL2DZ level of theory. Color coding: blue–titanium, red–oxygen, gray–carbon, white–hydrogen.

#### 3.1.2 The C–H Bond Activation in Adsorbed Methane (CH_4_)

The first step is the activation of one of the C–H bonds of the adsorbed CH_4_ by the Ti atom (**4,** Figure 3) via a 1,2-addition mechanism.^38–40^ The CH_3_ group is bound to the titanium atom, while the hydrogen atom from the activated C–H bond binds to the adjacent oxygen atom. The preliminary binding of methane to this early transition metal (Ti) occurs via sigma complex formation to the d_*z*_^2^ orbital of titanium. The cleavage of the C–H bond and transfer of the hydrogen atom arise from the donation of electron density from the metal–ligand dπ orbital to the C–H σ* orbital of the CH_4_. The transition state **4** (Figure 3) is a four-membered transition state involving CH_4_ carbon, C–H bonded hydrogen, a Ti atom attached to CH_4_, and oxygen adjacent to the methane-adsorbed Ti atom. This mechanism looks similar to σ-bond metathesis. The resulting Ti–C bond strength between the Ti atom and CH_3_ drives forward C–H activation of the C–H bond. The energy of activation (*E*_act_) for the C–H activation reaction is 148.8 kJ/mol. This is a low energy barrier indicating a forward reaction to form product **5** (Figure 3) where CH_3_ is bonded to the Ti atom and the hydrogen atom to the oxygen adjacent to the CH_3_-bonded Ti atom. The CH_3_ carbon attached to the Ti atom in the transition state has a charge of −1.0042 (C18), exhibiting a carbanion character (Figure S1, Table S1).^41,42^ In photocatalytic transformations, a hydrogen atom transfer to adjacent electronegative atoms/groups and the generation of carbanions are crucial intermediates. The product is slightly higher in energy compared to that of the reactant (Δ*E* = 25.0 kJ/mol). This means that the product is not stable and susceptible to further reactions. This is an encouraging result, as we want a less stable product for further methane coupling reaction to happen. A second methane interacts with a Ti atom adjacent to Ti-CH_3_, to form structure **6**. Structure **6** undergoes a second C–H activation reaction via transition state **7** (obtained from PES scan) with *E*_act_ = 303.83 kJ/mol (which can be lowered if full IRC are done) and Δ*E* = 35.60 kJ/mol to form two C–H activated products **8**. Here the substrate **6** is more stable than the product **8**; the reaction is not spontaneous, and the unstable product **8** is susceptible to a further forward coupling reaction.

**Figure 3:**
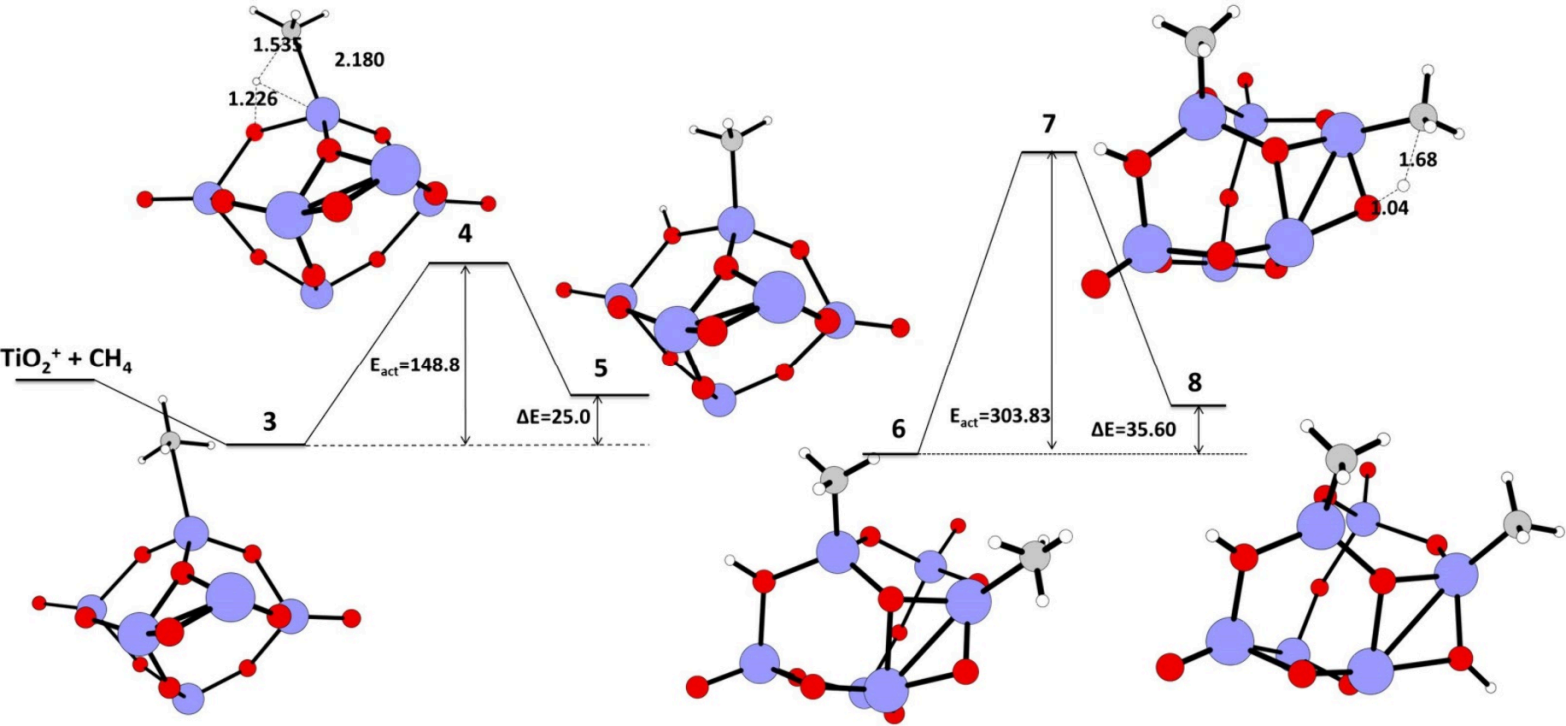
Schematic representation of the calculated IRC path for the first and second C–H activation reaction of methane on a TiO_2_ nano cluster. The reaction energy (Δ*E*) in kJ/mol, energy of activation (*E*_act_) in kJ/mol for the reactant, transition state, and product geometries, and the bond lengths (Å) in the transition state calculated at the UB3LYP/LANL2DZ level of theory. Color coding: blue–titanium, red–oxygen, gray–carbon, white–hydrogen.

#### 3.1.3 The C–C Coupling Reaction to Form Ethane on a TiO_2_ Nano Cluster

We studied the coupling reaction between two adjacent adsorbed CH_3_ on TiO_2_ nano cluster (structure **9**, Figure 4) to form ethane. The structure of **9** has two CH_3_ attached to two adjacent Ti atoms and two hydrogens on oxygen atoms adjacent to two Ti-CH_3_. The structure **9** undergoes a C–C coupling reaction via transition state **10** (Figure 4) with an *E*_act_ = 158.4 kJ/mol and Δ*E* = 36.9 kJ/mol for the C–C coupling reaction between two CH_3_ attached on two adjacent Ti atoms. Here in the transition state **10** there is C–H activation of one of the CH_3_ attached to the Ti atom and the C–C coupling between CH_2_ and the other adsorbed CH_3_. The two carbons involved in the coupling in the transition state have large negative charges on them, C18 (−0.8081) and C23 (−0.7311), demonstrating carbanion character (Figure S2, Table S2).^41–43^ The hydrogen (H26) transferred/attached to the Ti atom in the transition state **10** has a negative charge of −0.0247 (Figure S2, Table S2).^44^ Here, the carbanion generated during C–H bond activation drives the reaction forward to C–C bond formation and hydrogen atom abstraction by an adjacent electronegative atom. The subsequent product **11** (Figure 4) has CH_2_CH_3_ attached to a Ti atom, hydrogen attached to an adjacent Ti atom, and two hydrogens bonded to oxygen atoms. The CH_2_CH_3_ attached to the Ti atom in structure **11** (see Figure 5) grabs the hydrogen bonded to the adjacent Ti atom via a C–H formation reaction via transition state **13** to form intermediate **14**. The *E*_act_ for this C–H formation is 131.3 kJ/mol, and Δ*E* is 78.8 kJ/mol. The intermediate **14** has CH_3_–CH_3_ (ethane) attached to a Ti atom via weak bonding to one of the CH_3_ carbons in ethane. The energy needed for the final desorption of ethane from the TiO_2_ surface is −244.76 kJ/mol (the product preferentially detaches from the nano catalyst).

**Figure 4:**
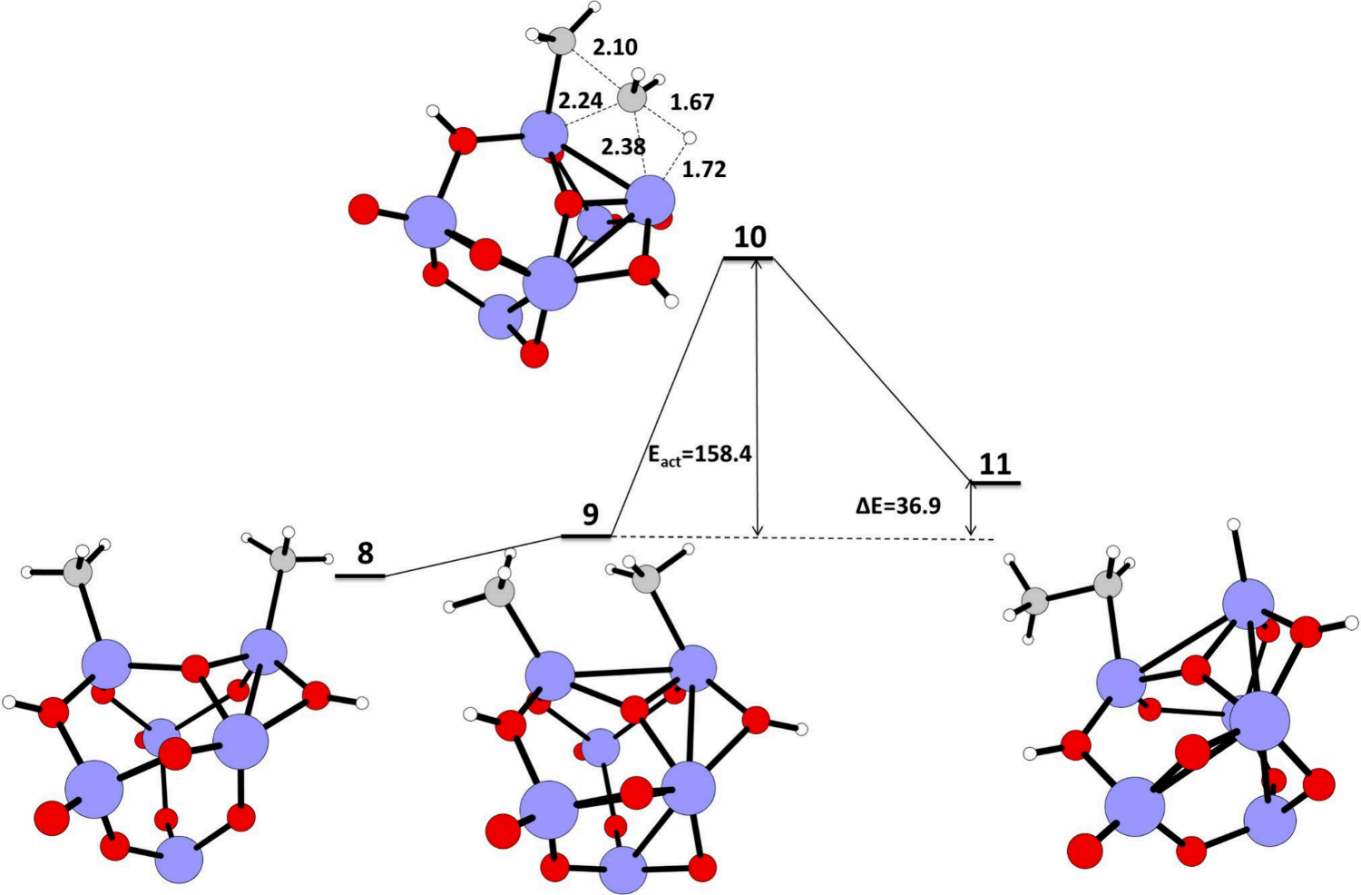
Schematic representation of the calculated IRC path for the C–C coupling reaction of methane on a TiO_2_ nano cluster. The reaction energy (Δ*E*) in kJ/mol, energy of activation (*E*_act_) in kJ/mol for the reactant, transition state, and product geometries, and the bond lengths in Å in the transition state calculated at the UB3LYP/LANL2DZ level of theory. Color coding: blue–titanium, red–oxygen, gray–carbon, white–hydrogen.

**Figure 5:**
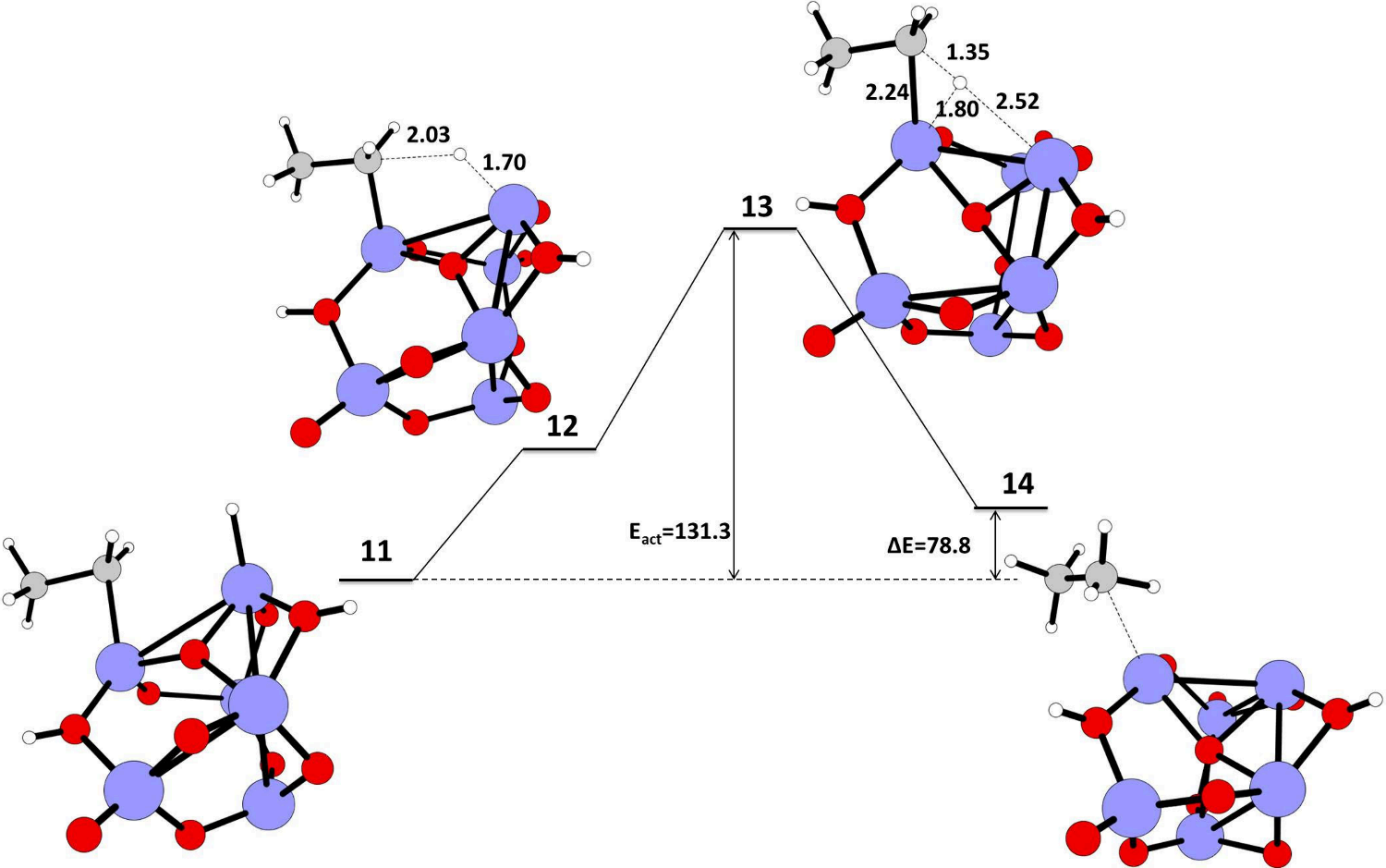
Schematic representation of the calculated IRC path for C–H formation reaction of methane on a TiO_2_ nano cluster. The reaction energy (Δ*E*), energy of activation (*E*_act_) and reactant, transition state, and product geometries, and the bond lengths of the transition state calculated at the UB3LYP/LANL2DZ level of theory. Color coding: blue–titanium, red–oxygen, gray–carbon, white–hydrogen.

Overall, the proposed reaction mechanism consists of a series of stages. The first step is methane adsorption on TiO_2_ (**3**), then C–H activation of adsorbed CH_4_ on TiO_2_ by the Ti atom (**4**) to give the product with CH_3_ bonded to the Ti atom and H on the adjacent oxygen atom (**5**). The next step can be desorption of a CH_3_ radical with an energy of desorption of 279.49 kJ/mol or the reaction can go to a second adsorption of CH_4_ adjacent to an adsorbed CH_4_ and undergo a C–C coupling reaction to form **11**, followed by a C–H formation reaction to give ethane bonded toTiO_2_ (**14**) and a final desorption of ethane from the TiO_2_ nano cluster. According to our calculations, the C–C coupling step is the rate limiting one.

### 3.2 Methane to Ethane Conversion on a Au_6_–TiO_2_ Nano Cluster

Combining TiO_2_ with appropriate cocatalysts, here Au_6_^–^, is another promising strategy to tune and optimize the performance of photocatalytic methane to ethane conversion. The Au–TiO_2_ cocatalysts can influence the performance via the three following aspects: (1) the cocatalysts can promote the solar-to-chemical conversion efficiency; (2) the cocatalysts can reduce the energy barrier of methane activation; (3) the adsorption of anionic Au_6_^–^ atoms/clusters on TiO_2_ donate electronic charge from the high-lying orbitals of the Au_6_^–^ nano clusters to the LUMO of the target molecules, reducing the barrier for the conversion of methane to ethane.^45–49^ The effect of attaching a gold nano cluster (Au_6_^–^) on aTiO_2_ cluster for adsorption of CH_4_ and formation to ethane is studied. The Au_6_^–^ is attached on TiO_2_ with two oxygen vacancies. The anionic Au_6_^–^ nano cluster with a triangular planar structure is bonded to a TiO_2_ nano cluster via triangular face sharing with TiO_2_. The resulting Au_6_TiO_2_ nano cluster has one unpaired electron and a positive charge. The Au–TiO_2_ nano cluster HOMO–LUMO gap is 2.18 eV (568 nm), which falls in the visible range wavelength of the electromagnetic spectrum. There are three unique binding positions of a gold nano cluster on TiO_2_ cluster (Figure S5). Out of the three possibilities, structure **15** in Figure 6, where the gold nano cluster is adjacent to the binding site of CH_4_ on a Ti atom is found to be the stable one. The C–H activation energy barrier with a Au_6_^–^ cluster on TiO_2_ is 155.6 kJ/mol, and the energy for the reaction is 27.4 kJ/mol, indicating a forward reaction. The reaction energy with a Au_6_^–^-containing TiO_2_ catalyst is only ∼2 kJ/mol higher compared to a bare TiO_2_ nano cluster as the catalyst. This may be due to reorganization energy compensation of the strain between Au and the TiO_2_ nano cluster interface in the case of Au_6_TiO_2_ compared to a bare TiO_2_ nano cluster.^50,51^ The CH_3_ carbon in transition state **16** has a high negative charge of −1.0084 (C17) with carbanion nature (Figure S3, Table S3).^41,42^ The resulting product **17** is higher in energy than the reactant, showing the feasibility for further reaction. The product **17** being less stable undergoes a further forward reaction.

**Figure 6:**
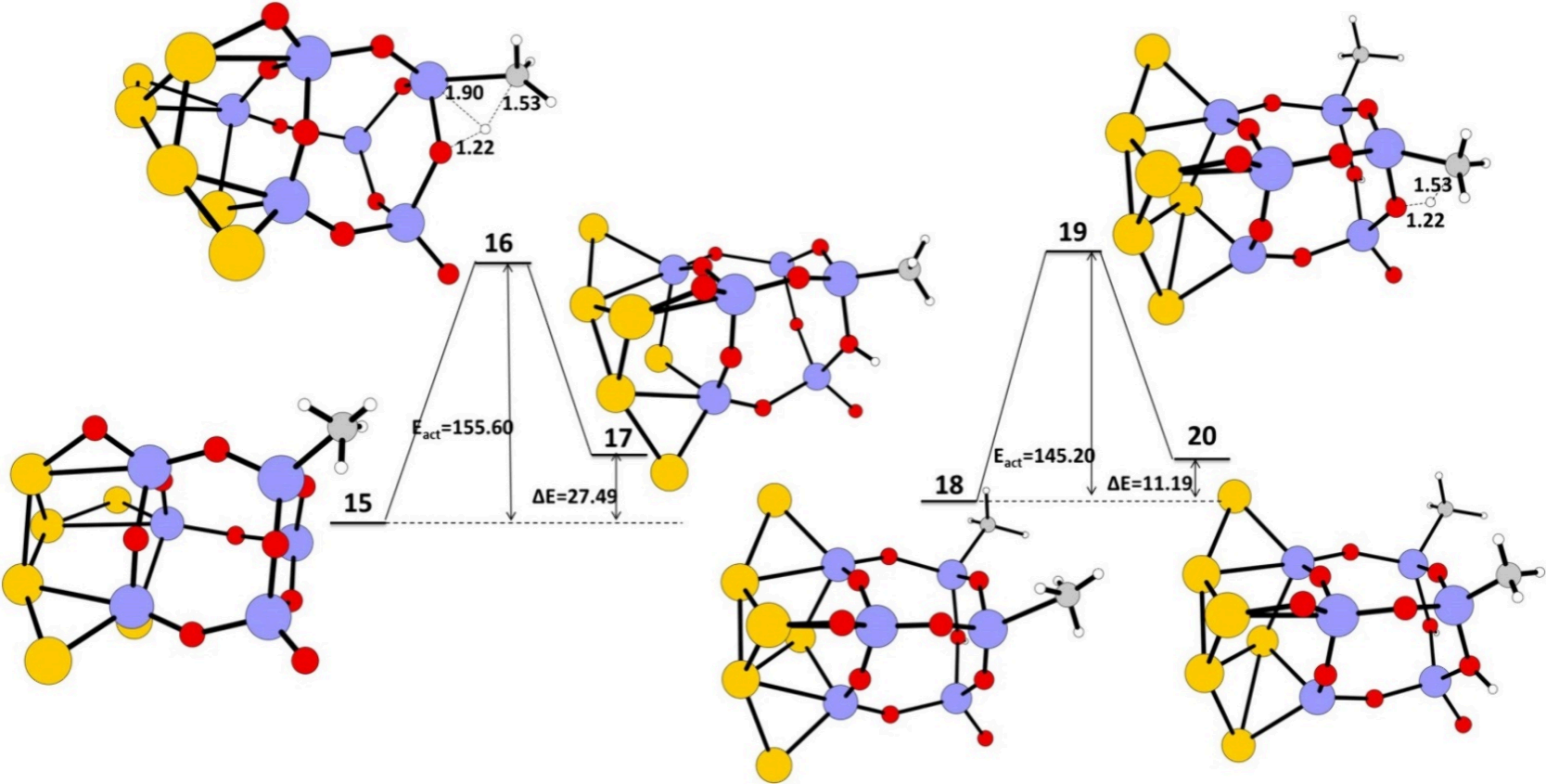
Schematic representation of the calculated IRC path for the first and second C–H activation reaction of methane on a Au_6_–TiO_2_ nano cluster. The reaction energy (Δ*E*) in kJ/mol, energy of activation (*E*_act_) in kJ/mol and reactant, transition state, and product geometries, and the bond lengths in Å in the transition state calculated at UB3LYP/LANL2DZ are given. Color coding: blue–titanium, red–oxygen, gray–carbon, white–hydrogen, yellow–gold.

The structure **17** interacts with a second CH_4_ to give structure **18**, which undergoes a second C–H activation via transition state **19** with an energy of activation, *E*_act_ = 145.20 kJ/mol, and reaction energy, Δ*E* = 11.19 kJ/mol, to give double C–H activated product **20**, Figure 6.

The C–C coupling reaction starts with structure **20** (Figure 7) with two CH_3_ attached to two Ti adjacent atoms and two hydrogen atoms to two oxygen atoms adjacent to the two Ti atoms. The structure **20** undergoes a C–C coupling reaction through the intermediate **21** to form the product **22** with CH_2_CH_3_ attached to one Ti atom and a hydrogen atom on an adjacent Ti atom. The CH_2_CH_3_ in **22** undergoes a hydrogen abstraction reaction of the hydrogen attached to the adjacent Ti atom to form a final ethane molecule weakly attached on TiO_2_ nano cluster **23**. The energy of activation and reaction energy for the C–C coupling reaction are 148.3 and 10.7 kJ/mol, respectively. The two carbons involved in the coupling in the transition state have large negative charges on them, C17 (−0.7428) and C29 (−0.7792), demonstrating carbanion character (Figure S4, Table S4).^41–43^ The hydrogen transferred/attached to the Ti atom in the transition state **21** has a negative charge of (H18) −0.1031 (Figure S4, Table S4). The reaction energy for the C–C coupling reaction in the Au–TiO_2_ nano cluster is found to be significantly less compared the bare TiO_2_ nano cluster. The introduction of a Au_6_^–^ cluster as a cocatalyst on a TiO_2_ nano cluster reduced the energy barrier and reaction energy for the C–C coupling reaction compared to C–C coupling reaction on a pure TiO_2_ nano cluster alone and thus improves the methane to ethane conversion. The final desorption energy of ethane from TiO_2_ is calculated to be −62.66 kJ/mol.

**Figure 7:**
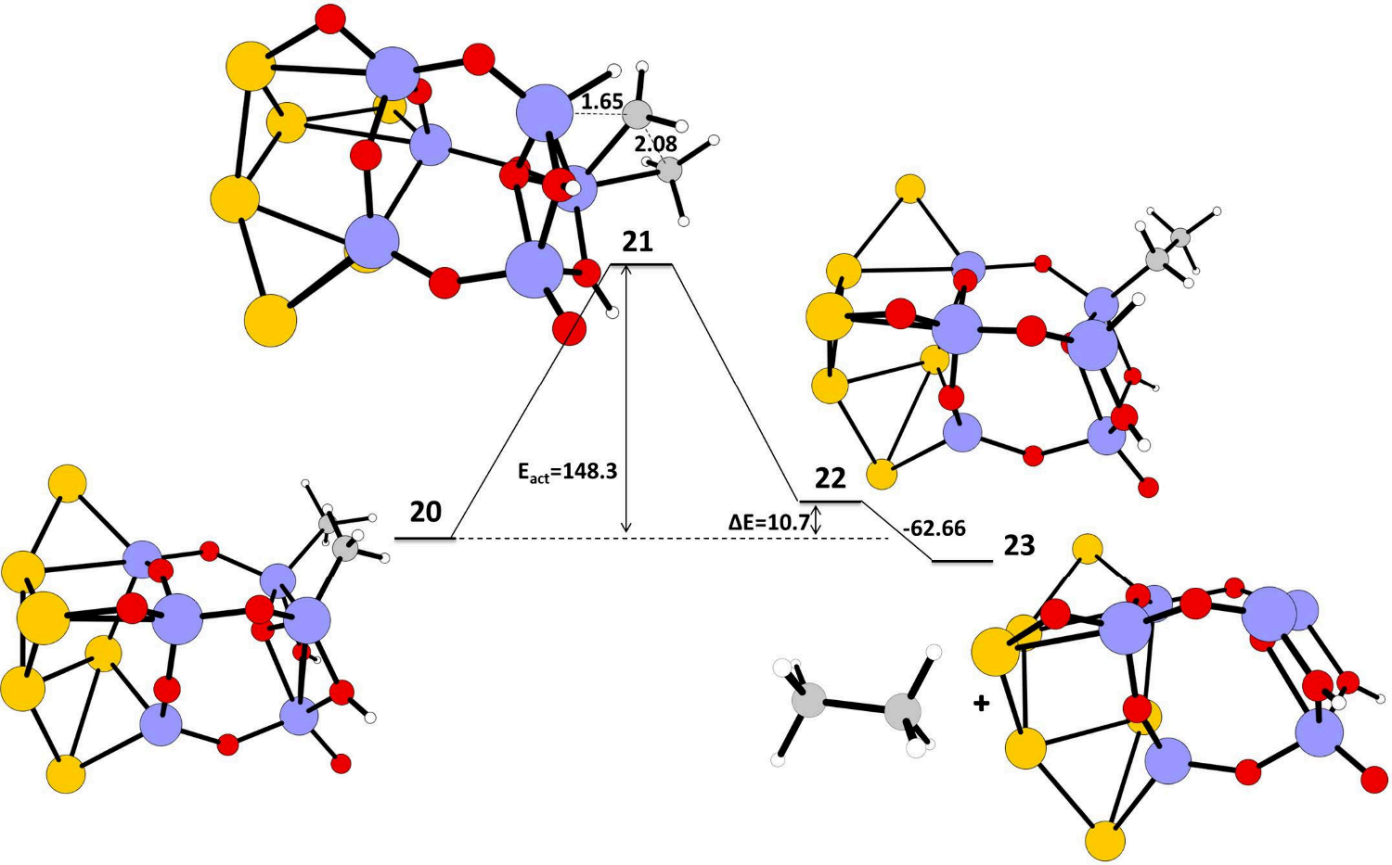
Schematic representation of the calculated IRC path for C–C coupling reaction of methane on a Au_6_–TiO_2_ nano cluster. The reaction energy (Δ*E*) in kJ/mol, energy of activation (*E*_act_) in kJ/mol and reactant, transition state, and product geometries, and the bond lengths in Å in the transition state calculated at UB3LYP/LANL2DZ are given. Color coding: blue–titanium, red–oxygen, gray–carbon, white–hydrogen, yellow–gold.

Our calculations shed light on the possible ways of ethane production via methane coupling on the surface of TiO_2_ and Au_6_–TiO_2_ nano clusters. The comparison of the energetics of the reaction indicates that the process occurring on a TiO_2_ or Au_6_–TiO_2_ system is similar—see Figure 8. The energies needed to split the first C–H bond in methane are lower in the case of the titania surface (148.8 kJ/mol on TiO_2_ vs 155.6 kJ/mol on Au_6_–TiO_2_). The activation of the second methane molecule is found to be easier on the gold–TiO_2_ system. The C–C coupling step is facilitated by the presence of gold (158.4 kJ/mol on TiO_2_ vs 148.3 kJ/mol on Au_6_–TiO_2_). The desorption of ethane is thermodynamically favored on the Au_6_–TiO_2_ system.

**Figure 8:**
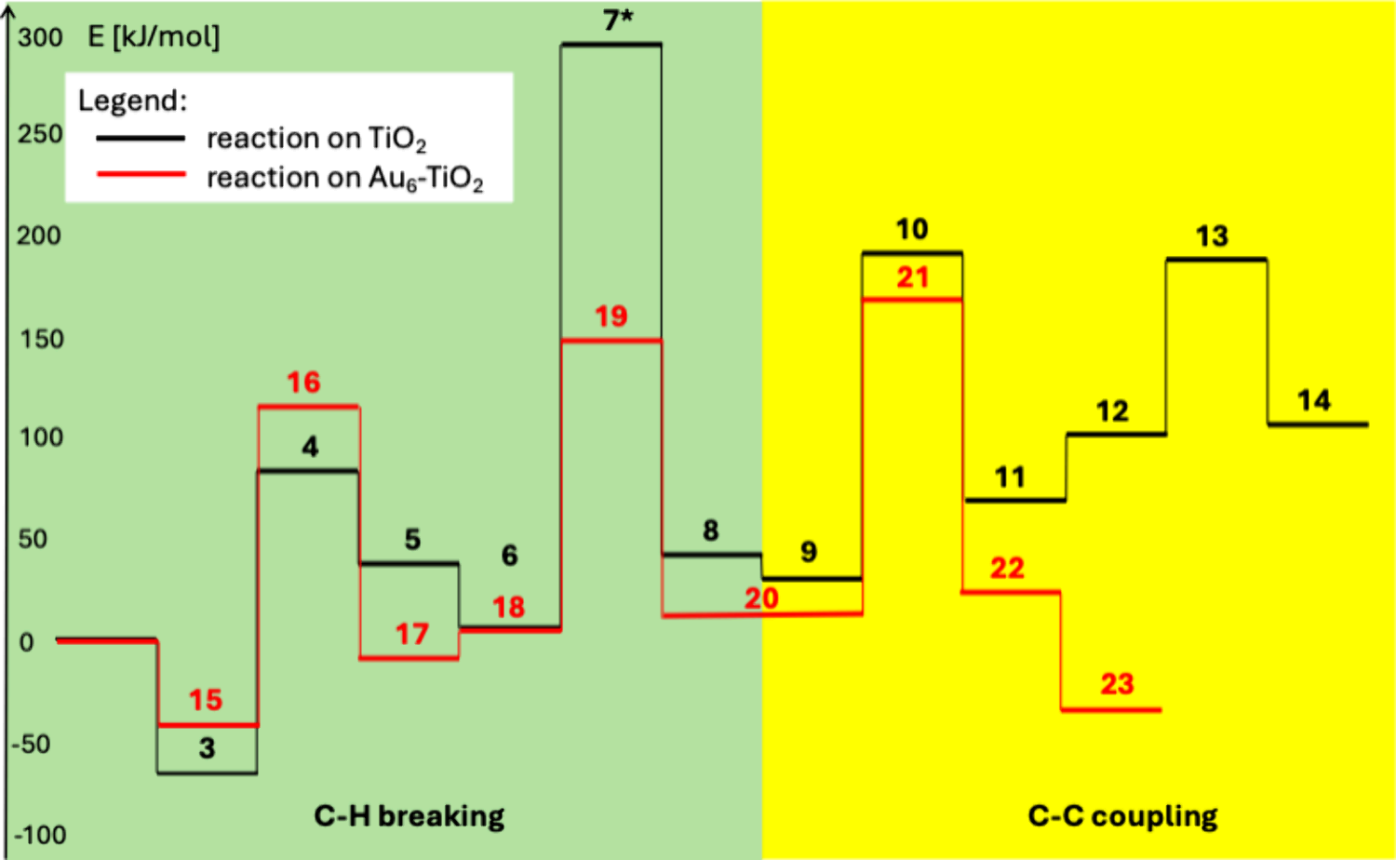
Schematic comparison of the methane coupling reaction pathways on TiO_2_ and Au_6_–TiO_2_ nano clusters calculated at UB3LYP/LANL2DZ level. *structure **7** is calculated form the potential energy surface scan.

### 3.3 Methane Activation by Photogenerated Reactive Oxygen Species

Recent discussion in the literature indicates that methane can also undergo activation in the gas phase under photocatalytic conditions.^52^ Our theoretical studies take into account this possibility too. The activation of the C–H bond in methane can occur under the influence of the so-called reactive oxygen species (ROS)–·OH, ·OOH, and ^1^O_2_, which are formed in the presence of photocatalysts from molecules present in the reaction environment (mainly O_2_ and H_2_O) under light adsorption. Our previous studies (MP2/6-31g*) indicate the possibility of this methane activation pathway under photocatalytic conditions: the calculated energy barrier for the C–H bond cleavage reaction in methane is equal to 64.4 kJ/mol in the case of the reaction with ·OH.^52^ In here, we consider also other free radicals which may be present in the reaction environment, ^1^O_2_ and ·OOH. The C–H bond can also be cleaved in the reaction with ^1^O_2_—the computed reaction barrier is 82.8 kJ/mol. The reaction with ·OOH is not favorable (activation barrier amounts to 308.8 kJ/mol). The concentration of ROS and their relatively short lifetimes mean that the reaction paths with ROS are only one of the possibilities, not excluding reactions occurring on the photocatalyst surface.

The conducted research is a voice in the long-standing discussion on the mechanism of the methane coupling reaction, initiated by Lunsford^53^ who conducted work on methane coupling under thermal catalysis on Na–W–Mo systems and postulated its homogeneous–heterogeneous mechanism. According to him, the C–H bond activation reaction takes place on the catalyst surface (heterogeneous step), methyl radicals are detached from the surface, and then, the C–C bond formation takes place in the gas phase (homogeneous step). In the case of the reactions studied herein, methane activation can occur either in the gas phase or on the photocatalyst surface, whereas the coupling of two ·CH_3_ radicals takes place on the surface.

## 4 Conclusions

The TiO_2_ nano clusters with a band gap that falls in the solar spectrum have the ability to harness light for efficient photocatalytic conversion of methane to ethane. The different reaction steps involved in the mechanistic pathway of methane to ethane conversion are studied. The reaction energies (Δ*E*) and energy of activation (*E*_act_) calculated from the reaction mechanism for different steps confirm the successful conversion of methane to ethane under normal conditions on TiO_2_ and Au_6_–TiO_2_ nano clusters. In the C–H activation reaction on the Au_6_–TiO_2_ nano cluster, the reaction energy (Δ*E*) is almost the same as that on the bare TiO_2_ nano cluster. The second C–H activation of the methane molecule is found to be easier on the gold–TiO_2_ system than on the bare TiO_2_ nano cluster. The introduction of a gold nano (Au_6_^–^) cluster as a cocatalyst with a TiO_2_ nano cluster reduces the energy needed for the C–C coupling reaction step in the methane to ethane formation reaction but slightly increases the barrier for the C–H bond activation. The Au_6_^–^ species donating electrons to the C–H bond leads to a weakening of the C–H bond and C–H activation. Taking into account that the methane activation can take place also in the gas phase, in the reaction with the reactive oxygen species formed at the surface of the photocatalyst, the gold cocatalyst has a beneficial impact on the overall catalytic efficiency of the process. The sp^3^-hybridized CH_3_ carbon involved in the transition states of C–H activation and C–C coupling shows carbanion character. The carbanion generation during transition state of the C–C bond formation reaction drives the reaction forward to form ethane, thereby increasing selectivity and yield of the product. The final desorption energy is lower in case of the Au_6_–TiO_2_ nano cluster compared to TiO_2_ nano cluster. This mechanistic study of the methane to ethane conversion reaction on TiO_2_ and Au_6_–TiO_2_ nano clusters sheds light on designing TiO_2_-based nanocluster catalysts for efficient photocatalytic yield. A deep understanding of the key catalytic steps involved in the methane to ethane conversion reaction helps in tuning high yield ethane formation reaction and reducing unwanted reaction products.

## Supplementary Material



## Notes

NBO charges and geometry structures for structure **4** (Table S1, Figure S1), structure **10** (Table S2, Figure S2); structure **16** (Table S3, Figure S3); and structure **21** (Table S4, Figure S4) as well as the Cartesian coordinates of the optimized geometries of structures calculated at UB3LYP/LANL2DZ level of theory (PDF)
