# Supplementary material for: A Mechanistic Study on the Photocatalytic Conversion of Methane to Ethane on TiO2 and Au–TiO2 Nano Clusters
Source: J Phys Chem A. 2025 Sep 26;129(40):9149–57. doi: 10.1021/acs.jpca.5c03782 (PMC12516712; doi:10.1021/acs.jpca.5c03782)
Supplement: Supplementary file 1 [file jp5c03782_si_001.pdf]

Supplementary information for:

A mechanistic study on the photo catalytic conversion of methane to ethane on TiO_2 and Au-TiO_2 nano clusters

Vidya Kaipanchery, Dorota Rutkowska-Zbik

Jerzy Haber Institute of Catalysis and Surface Chemistry PAS, ul. Niezapominajek 8, 30-239 Kraków, Poland

e-mail: dorota.rutkowska-zbik@ikifp.edu.pl; ORCID: 0000-0001-9323-1710

Table S1: NBO Charges for structure **4**:

1	Ti	1.448448
2	Ti	1.350228
3	Ti	1.437663
4	Ti	1.368820
5	Ti	1.245885
6	Ti	1.278885
7	O	-0.688279
8	O	-0.703488
9	O	-0.952024
10	O	-0.752437
11	O	-0.709000
12	O	-0.718523
13	O	-0.668554
14	O	-0.828712
15	O	-0.319892
16	O	-0.327671
17	O	-0.727427
18	C	-1.004175
19	H	0.305895
20	H	0.272847
21	H	0.298014
22	H	0.393498

Figure S1: Structure **4** labelled and numbered.

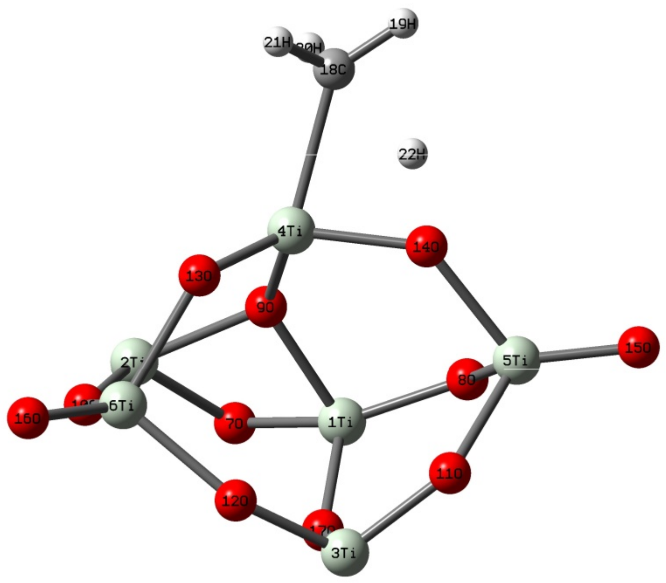

Table S2: NBO Charge for structure **10**.

1	Ti	1.389042
2	Ti	1.225373
3	Ti	1.274572
4	Ti	1.378813
5	Ti	1.121674
6	Ti	1.191687
7	O	-0.877460
8	O	-0.657599
9	O	-0.928209
10	O	-0.640129
11	O	-0.704931
12	O	-0.765155
13	O	-0.639641
14	O	-0.921290
15	O	-0.339551
16	O	-0.330111
17	O	-0.636073
18	C	-0.808077
19	H	0.279079
20	H	0.316820
21	H	0.296261
22	H	0.463950
23	C	-0.731145
24	H	0.321497
25	H	0.297137
26	H	-0.024693
27	H	0.448158

Figure S2: Structure **10** labelled and numbered.

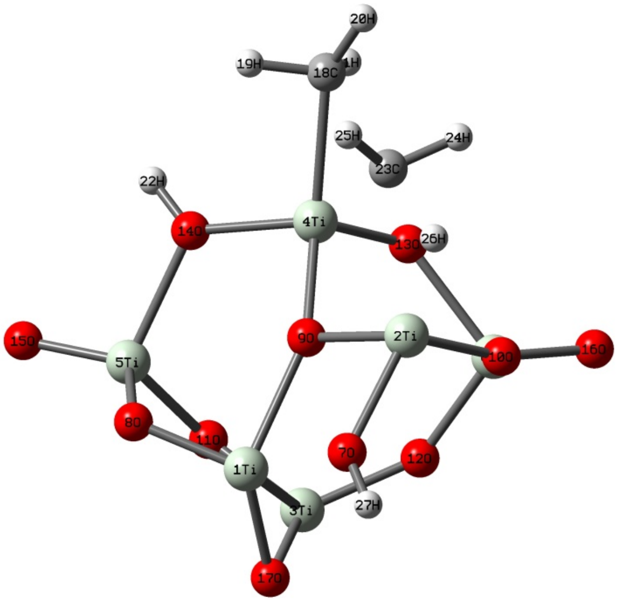

Table S3: NBO Charge for structure **16**.

1	Ti	1.200861
2	Ti	1.447844
3	Ti	1.431685
4	Ti	1.440082
5	Ti	1.205359
6	Ti	1.283437
7	O	-0.729020
8	O	-0.754426
9	O	-0.747977
10	O	-0.735333
11	O	-0.716186
12	O	-0.741036
13	O	-0.738591
14	O	-0.861421
15	O	-0.346823
16	O	-0.476026
17	C	-1.008339
18	H	0.284633
19	H	0.296280
20	H	0.281059
21	H	0.391598
22	Au	0.499707
23	Au	0.475203
24	Au	-0.716764
25	Au	-0.359823
26	Au	0.070071
27	Au	-0.376054

Figure S3: Structure **16** labelled and numbered.

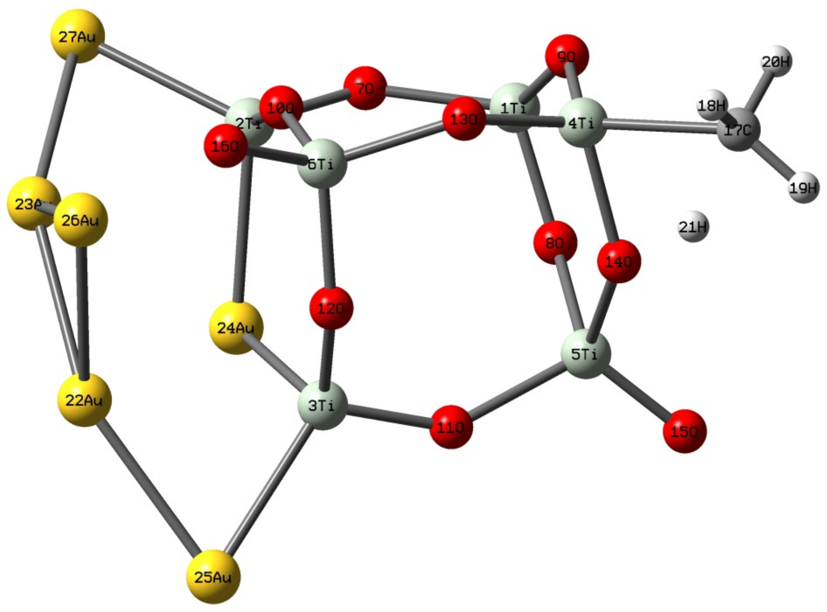

Table S4: NBO Charges for structure **21**.

1	Ti	1.261759
2	Ti	1.455918
3	Ti	1.149316
4	Ti	1.332395
5	Ti	1.160701
6	Ti	1.300210
7	O	-0.728437
8	O	-0.891523
9	O	-0.768224
10	O	-0.722378
11	O	-0.768703
12	O	-0.768135
13	O	-0.665009
14	O	-0.893007
15	O	-0.363950
16	O	-0.479576
17	C	-0.742818
18	H	-0.103063
19	H	0.306357
20	H	0.313132
21	H	0.456962
22	Au	0.433103
23	Au	0.407805
24	Au	-0.526746
25	Au	-0.308761
26	Au	0.012586
27	Au	-0.401198
28	H	0.454160

29	C	-0.779166
30	H	0.302654
31	H	0.299726
32	H	0.263910

Figure S4: Structure **21** labelled and numbered.

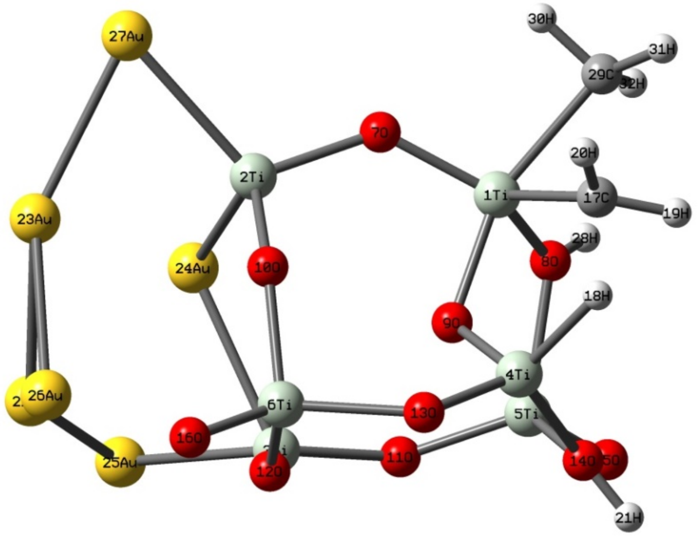

Optimized geometries of structures calculated at UB3LYP/LANL2DZ level of theory

TiO_2 cluster with single oxygen vacancy and +1 charge

1	22	0	0.598064	1.713422	-0.792163
2	22	0	-2.107293	2.042192	-0.026558
3	22	0	0.580191	-1.453705	-1.916618
4	22	0	-0.038785	0.045481	2.207268
5	22	0	2.835745	-0.308987	0.388257
6	22	0	-2.245987	-1.425061	0.121793
7	8	0	-0.792400	2.890101	-1.037779
8	8	0	2.246122	1.440038	-0.225035
9	8	0	-0.370160	1.303606	0.882196
10	8	0	-2.738587	0.412564	-0.247350
11	8	0	1.985946	-1.506781	-0.906050
12	8	0	-0.830697	-2.000652	-1.080791
13	8	0	-1.219118	-1.127376	1.775542
14	8	0	1.562899	-0.471408	1.866569

15	8	0	4.409665	-0.526481	0.699157
16	8	0	-3.505005	-2.434714	0.249468
17	8	0	0.291013	0.334416	-1.926367

Structure 1

1	22	0	-1.445645	-0.527620	1.764621
2	22	0	0.584354	1.617774	1.369873
3	22	0	0.461218	-2.028451	0.204918
4	22	0	-0.075665	1.122466	-1.400200
5	22	0	-2.265204	-1.210809	-1.327399
6	22	0	2.902156	0.067599	-0.420616
7	8	0	-0.728199	0.962710	2.514690
8	8	0	-2.660281	-0.720768	0.462702
9	8	0	-0.075194	-0.040250	0.297664
10	8	0	2.217813	1.089200	1.128399
11	8	0	-0.761050	-2.304742	-1.078013
12	8	0	2.133727	-1.679953	-0.099414
13	8	0	1.545388	0.651756	-1.746186
14	8	0	-0.012296	2.447374	-0.117870
15	8	0	-1.527131	0.265481	-2.102099
16	8	0	4.470618	0.178291	-0.789891
17	8	0	-0.422656	-1.993929	1.848628
18	6	0	-3.697459	3.023807	-0.675380
19	1	0	-2.726342	3.374252	-0.306524
20	1	0	-3.601462	2.668535	-1.708325
21	1	0	-4.072674	2.216006	-0.035927
22	1	0	-4.407393	3.855891	-0.652114

Structure 2

1	22	0	3.217255	-0.961269	0.038190
2	22	0	1.005254	1.121829	1.463169
3	22	0	0.099000	-0.761740	-1.309822
4	22	0	-1.773183	0.548987	1.303870
5	22	0	-2.753978	-2.306239	-0.241907
6	22	0	-0.575318	2.421539	-1.079443
7	8	0	2.618640	0.116874	1.303983
8	8	0	-0.115304	-0.113996	0.465323
9	8	0	0.866488	2.373299	0.277375
10	8	0	-1.311089	-1.859632	-1.343214
11	8	0	-0.162336	0.788730	-2.060190
12	8	0	-2.000002	1.785082	0.104272
13	8	0	-0.467247	1.157922	2.517847
14	8	0	-2.763855	-0.928987	1.001165
15	8	0	-2.524956	-3.746188	0.466328
16	8	0	-0.800780	3.788247	-1.916832
17	8	0	1.920195	-1.267535	-1.120237
18	6	0	5.484971	-1.802241	-0.138085

19	1	0	4.899025	-2.592001	0.372343
20	1	0	5.189647	-1.627065	-1.187783
21	1	0	5.613126	-0.880760	0.455512
22	1	0	6.491673	-2.225614	-0.207396

Structure 3

1	22	0	-0.621555	-0.301872	1.919433
2	22	0	2.089315	0.440793	1.974257
3	22	0	-0.644880	-2.486386	-0.634268
4	22	0	0.080036	1.879355	-0.700188
5	22	0	-2.833192	0.125655	-0.396561
6	22	0	2.226967	-0.640996	-1.316285
7	8	0	0.756313	-0.166834	3.129472
8	8	0	-2.262235	0.162775	1.463927
9	8	0	0.388585	1.089922	0.962884
10	8	0	2.719236	-0.363930	0.539584
11	8	0	-2.030900	-1.535310	-1.053114
12	8	0	0.782254	-1.937334	-1.441311
13	8	0	1.259460	1.034264	-1.631372
14	8	0	-1.536770	1.414950	-1.070203
15	8	0	-4.398213	0.388495	-0.725168
16	8	0	3.486450	-0.908934	-2.299945
17	8	0	-0.349142	-1.865580	1.038843
18	6	0	0.293845	4.285249	-0.989481
19	1	0	1.173763	3.861187	-1.502279
20	1	0	-0.673960	4.026922	-1.450414
21	1	0	0.302769	4.172154	0.109050
22	1	0	0.386851	5.364272	-1.148790

Structure 4

1	22	0	0.715187	0.095874	1.893584
2	22	0	-2.025568	-0.597600	2.044584
3	22	0	0.658276	2.415688	-0.506057
4	22	0	-0.219953	-1.781996	-0.874102
5	22	0	2.818665	-0.265700	-0.576161
6	22	0	-2.358231	0.739787	-1.134801
7	8	0	-0.600296	-0.109204	3.152895
8	8	0	2.296772	-0.412540	1.280670
9	8	0	-0.401099	-1.150643	0.864603
10	8	0	-2.734451	0.323195	0.720607
11	8	0	1.956070	1.414908	-1.079308
12	8	0	-0.844697	1.962365	-1.225676
13	8	0	-1.459338	-0.919997	-1.695402
14	8	0	1.533091	-1.553272	-1.331083
15	8	0	4.389828	-0.438734	-0.927054
16	8	0	-3.651308	1.184479	-2.001292
17	8	0	0.468460	1.739136	1.159869
18	6	0	0.226254	-3.840252	-1.437996

19	1	0	1.088158	-4.412185	-1.819171
20	1	0	-0.044542	-4.335172	-0.489855
21	1	0	-0.570668	-3.993216	-2.180817
22	1	0	1.291041	-2.738914	-1.528073

Structure 5

1	22	0	0.701344	0.083841	1.890527
2	22	0	-2.017514	-0.605661	2.033089
3	22	0	0.650938	2.418227	-0.502063
4	22	0	-0.183195	-1.820577	-0.895961
5	22	0	2.839242	-0.214458	-0.531731
6	22	0	-2.351388	0.718505	-1.136862
7	8	0	-0.598743	-0.099254	3.156505
8	8	0	2.305507	-0.416827	1.290469
9	8	0	-0.380055	-1.149623	0.860832
10	8	0	-2.731767	0.326890	0.725455
11	8	0	1.962731	1.430561	-1.083967
12	8	0	-0.844022	1.957295	-1.227637
13	8	0	-1.453575	-0.951964	-1.676635
14	8	0	1.656391	-1.518598	-1.459507
15	8	0	4.390916	-0.439163	-0.944794
16	8	0	-3.641820	1.178277	-2.000502
17	8	0	0.470574	1.736934	1.157243
18	6	0	-0.204156	-3.826910	-1.292569
19	1	0	0.829246	-4.071653	-1.586504
20	1	0	-0.451527	-4.430005	-0.405480
21	1	0	-0.889832	-4.085079	-2.109249
22	1	0	2.051194	-2.027375	-2.199151

Structure 6

1	22	0	-1.184613	1.742681	-1.317185
2	22	0	1.975481	0.633871	-1.318442
3	22	0	-1.552436	1.517239	1.373337
4	22	0	0.686062	-2.184760	-0.066065
5	22	0	-2.635664	-0.981372	-0.355319
6	22	0	1.611608	0.377572	2.024837
7	8	0	0.644649	1.668395	-1.825942
8	8	0	-2.131999	0.278112	-1.677998
9	8	0	1.464701	-1.098685	-1.340214
10	8	0	2.423194	0.948035	0.317356
11	8	0	-2.517079	0.014781	1.230489
12	8	0	0.039121	1.349278	2.178643
13	8	0	1.086526	-1.408971	1.423276
14	8	0	-1.222112	-2.455017	-0.318354
15	8	0	-4.052825	-1.770151	-0.607949
16	8	0	2.601655	0.441155	3.326692
17	8	0	-1.843247	2.757773	0.033458
18	6	0	1.594119	-4.018982	-0.065375

19	1	0	1.414577	-4.513818	-1.033388
20	1	0	2.675619	-3.910383	0.095487
21	1	0	1.179299	-4.626310	0.753844
22	1	0	-1.623414	-3.341030	-0.435328
23	6	0	3.885583	1.035399	-2.826863
24	1	0	4.737507	1.229621	-3.484083
25	1	0	3.104576	1.741448	-3.151300
26	1	0	4.263200	1.228528	-1.809576
27	1	0	3.620116	-0.019325	-3.003440

Structure 7

1	22	0	-0.378256	-1.793459	-1.186657
2	22	0	2.339702	-1.077196	-0.174130
3	22	0	-1.103375	1.442244	-1.985807
4	22	0	-0.103367	-0.121289	1.992892
5	22	0	-2.991326	-0.297404	0.268257
6	22	0	1.585653	2.179459	0.103517
7	8	0	1.599107	-2.144106	-1.565528
8	8	0	-1.968580	-1.757539	-0.395622
9	8	0	0.650004	-1.225261	0.657737
10	8	0	2.686075	0.598397	-0.362077
11	8	0	-2.423528	1.142491	-0.867968
12	8	0	0.182316	2.312906	-1.184712
13	8	0	0.658214	1.379082	1.623180
14	8	0	-2.047882	-0.029706	2.033562
15	8	0	-4.591788	-0.497543	0.449537
16	8	0	2.395712	3.552875	0.389798
17	8	0	-0.476685	-0.188447	-2.211551
18	6	0	0.622192	-0.658432	3.808138
19	1	0	0.277271	-1.681174	4.029502
20	1	0	1.717132	-0.609496	3.869455
21	1	0	0.210302	0.048569	4.546517
22	1	0	-2.590724	0.142245	2.831613
23	6	0	3.796874	-2.551558	-0.403437
24	1	0	4.551680	-2.347339	0.378266
25	1	0	3.498753	-3.600064	-0.234904
26	1	0	4.365127	-2.466930	-1.345164
27	1	0	2.473649	-2.682861	-1.431903

Structure 8

1	22	0	-0.553851	0.908852	1.866633
2	22	0	2.315993	0.844839	0.983722
3	22	0	-1.281653	-2.223239	0.936932
4	22	0	0.092048	1.084965	-1.607348
5	22	0	-2.968815	0.475872	-0.345053
6	22	0	1.641550	-1.862408	-0.947104
7	8	0	1.391226	1.056306	2.667045
8	8	0	-2.068671	1.382054	1.062136

9	8	0	0.658986	1.298718	0.187669
10	8	0	2.603203	-0.724233	0.324788
11	8	0	-2.466804	-1.354100	-0.018687
12	8	0	0.111969	-2.586777	-0.040643
13	8	0	0.833944	-0.419331	-1.987565
14	8	0	-1.838118	1.093867	-1.893623
15	8	0	-4.548804	0.773052	-0.568627
16	8	0	2.534981	-2.948272	-1.753214
17	8	0	-0.742349	-0.968181	2.064919
18	6	0	0.995529	2.446208	-2.806710
19	1	0	0.613061	3.445951	-2.545082
20	1	0	2.089164	2.427879	-2.711919
21	1	0	0.738445	2.200746	-3.849368
22	1	0	-2.281415	1.360187	-2.726411
23	6	0	3.903826	2.040439	0.593503
24	1	0	4.259867	1.937008	-0.440734
25	1	0	3.643139	3.090046	0.800368
26	1	0	4.720814	1.724372	1.265520
27	1	0	1.668330	1.033720	3.602086

Structure 9

1	22	0	-1.155488	-0.713794	1.635121
2	22	0	1.850539	-0.077450	1.705574
3	22	0	-1.424877	-1.881314	-0.739408
4	22	0	0.851014	1.824401	-0.493844
5	22	0	-2.567946	1.124165	-0.460975
6	22	0	1.942201	-1.202061	-1.373197
7	8	0	0.602822	-0.997616	2.879049
8	8	0	-2.363068	0.558637	1.343189
9	8	0	0.387342	0.551248	0.875220
10	8	0	2.593352	-1.047744	0.499573
11	8	0	-2.348818	-0.482581	-1.399545
12	8	0	0.292108	-2.026451	-1.280515
13	8	0	1.594893	0.748576	-1.599730
14	8	0	-0.964801	2.338151	-0.871808
15	8	0	-3.892963	2.018625	-0.833969
16	8	0	2.981889	-1.887335	-2.429955
17	8	0	-1.774277	-2.310854	1.030151
18	6	0	1.757748	3.672241	-0.690135
19	1	0	1.304930	4.413541	-0.011083
20	1	0	2.845483	3.690195	-0.569259
21	1	0	1.528079	3.943982	-1.737737
22	1	0	-1.220730	3.183612	-1.293021
23	6	0	2.885350	1.756656	1.576021
24	1	0	3.493613	1.879931	0.670992
25	1	0	2.400187	2.705322	1.836492
26	1	0	3.618549	1.549359	2.386079
27	1	0	0.632360	-1.671500	3.580890

Structure 10

1	22	0	-1.199726	-0.769080	1.619761
2	22	0	1.838556	-0.071835	1.696506
3	22	0	-1.459665	-1.931148	-0.732079
4	22	0	0.836003	1.708485	-0.386080
5	22	0	-2.595975	1.086271	-0.455437
6	22	0	1.908062	-1.261395	-1.357771
7	8	0	0.582688	-1.048723	2.810426
8	8	0	-2.411915	0.509348	1.343782
9	8	0	0.272295	0.428948	0.839698
10	8	0	2.572387	-1.079587	0.525816
11	8	0	-2.377549	-0.521170	-1.390377
12	8	0	0.267236	-2.080136	-1.279130
13	8	0	1.524577	0.689106	-1.574100
14	8	0	-0.977025	2.325638	-0.813498
15	8	0	-3.922974	1.977359	-0.836374
16	8	0	2.949548	-1.929697	-2.425417
17	8	0	-1.823794	-2.377798	1.026319
18	6	0	2.005028	3.531911	-0.436539
19	1	0	1.173550	4.172200	-0.098413
20	1	0	2.940293	4.020490	-0.168009
21	1	0	2.020753	3.396560	-1.533959
22	1	0	-1.252977	3.145622	-1.268562
23	6	0	2.557939	2.086424	0.987425
24	1	0	3.569724	1.994627	0.585912
25	1	0	2.501242	2.861863	1.755666
26	1	0	3.030305	1.058765	2.215272
27	1	0	0.682911	-1.770740	3.457423

Structure 11

1	22	0	-1.161729	-0.706248	1.622655
2	22	0	1.894614	-0.158258	1.785953
3	22	0	-1.417237	-1.872605	-0.737193
4	22	0	0.787706	1.714137	-0.426088
5	22	0	-2.571372	1.147652	-0.471305
6	22	0	1.963805	-1.231333	-1.348776
7	8	0	0.602614	-1.160099	2.765320
8	8	0	-2.366203	0.573183	1.324435
9	8	0	0.385936	0.574673	0.958514
10	8	0	2.655183	-1.033008	0.523670
11	8	0	-2.322540	-0.460646	-1.403914
12	8	0	0.307431	-2.040380	-1.257938
13	8	0	1.526264	0.685333	-1.585086
14	8	0	-0.983590	2.437781	-0.835068
15	8	0	-3.916584	2.015550	-0.832934
16	8	0	2.987760	-1.894936	-2.434731
17	8	0	-1.793179	-2.303156	1.023354
18	6	0	2.245442	3.761137	-0.364470

19	1	0	1.353580	4.360573	-0.121121
20	1	0	3.099200	4.442453	-0.263645
21	1	0	2.233640	3.497090	-1.440591
22	1	0	-1.293356	3.299304	-1.175704
23	6	0	2.419317	2.529009	0.569426
24	1	0	3.393327	2.053563	0.420967
25	1	0	2.293571	2.812928	1.618763
26	1	0	3.000166	0.632685	2.757435
27	1	0	0.659809	-1.930445	3.360375

Structure 12

1	22	0	-1.151992	-1.141161	1.461644
2	22	0	1.799149	-0.423637	1.807217
3	22	0	-1.131006	-1.908837	-1.096415
4	22	0	0.581505	1.747096	-0.137564
5	22	0	-2.709213	0.838372	-0.432388
6	22	0	2.200806	-0.798357	-1.418172
7	8	0	0.517082	-1.520519	2.775148
8	8	0	-2.457719	0.057798	1.285269
9	8	0	0.334860	0.233866	0.929881
10	8	0	2.767750	-0.954906	0.489873
11	8	0	-2.201626	-0.546475	-1.589947
12	8	0	0.614557	-1.755039	-1.524078
13	8	0	1.605615	1.085506	-1.356962
14	8	0	-1.257880	2.290324	-0.556373
15	8	0	-4.150637	1.565140	-0.730054
16	8	0	3.312283	-1.191999	-2.549263
17	8	0	-1.529478	-2.663386	0.550840
18	6	0	1.397303	4.199492	0.139068
19	1	0	0.489424	4.690822	0.510228
20	1	0	2.207432	4.938851	0.152257
21	1	0	1.258277	3.980887	-0.943080
22	1	0	-1.601648	3.166404	-0.818766
23	6	0	1.786529	2.939960	0.988990
24	1	0	2.852733	2.721218	0.920538
25	1	0	1.476130	3.041853	2.032160
26	1	0	2.297036	1.171721	2.106384
27	1	0	0.515709	-2.247440	3.422211

Structure 13

1	22	0	-1.480334	-0.772287	1.508330
2	22	0	1.002072	-0.307291	1.935271
3	22	0	-1.264630	-1.987635	-1.355803
4	22	0	1.071215	1.603945	-0.228264
5	22	0	-2.268074	1.196123	-0.632235
6	22	0	2.141940	-1.428519	-0.844361
7	8	0	-0.469816	-0.655161	3.243426
8	8	0	-2.690539	0.511096	1.077784

9	8	0	-0.162431	0.279061	0.568740
10	8	0	1.997514	-1.491923	1.066725
11	8	0	-1.898657	-0.404096	-1.717271
12	8	0	0.460110	-2.074018	-1.569289
13	8	0	2.059668	0.480708	-1.135665
14	8	0	-0.692406	2.423901	-0.804589
15	8	0	-3.494720	2.071744	-1.276168
16	8	0	3.467038	-2.165974	-1.463716
17	8	0	-1.639198	-2.267131	0.370588
18	6	0	2.191785	3.871479	-0.500041
19	1	0	1.396054	4.561930	-0.199309
20	1	0	3.047486	4.460514	-0.847608
21	1	0	1.862426	3.335146	-1.420476
22	1	0	-0.835857	3.234494	-1.328619
23	6	0	2.631992	2.937228	0.663787
24	1	0	3.589768	2.458615	0.435071
25	1	0	2.741924	3.515958	1.585306
26	1	0	1.986044	1.926771	1.290377
27	1	0	-0.671156	-0.706727	4.193646

Structure 14

1	22	0	-1.220222	-1.632171	1.215753
2	22	0	1.044908	-0.426872	1.779593
3	22	0	-0.268888	-1.478439	-1.428227
4	22	0	0.352991	1.636609	0.123535
5	22	0	-2.706790	0.402366	-0.680279
6	22	0	2.786723	-0.428685	-0.825141
7	8	0	-0.196718	-1.465265	2.932631
8	8	0	-2.681495	-0.631348	0.935196
9	8	0	-0.172447	-0.247453	0.167308
10	8	0	2.444681	-1.111695	0.970798
11	8	0	-1.655874	-0.530540	-2.008588
12	8	0	1.433880	-1.274023	-1.888495
13	8	0	2.015403	1.295376	-0.339690
14	8	0	-1.587697	2.039084	-0.403783
15	8	0	-4.194557	0.883134	-1.159277
16	8	0	4.322700	-0.443794	-1.388851
17	8	0	-0.757865	-2.744211	-0.184584
18	6	0	1.004500	4.361047	0.021172
19	1	0	0.733140	5.370277	-0.309833
20	1	0	2.071195	4.342441	0.255411
21	1	0	0.857598	3.721986	-0.871919
22	1	0	-2.018461	2.892005	-0.601496
23	6	0	0.134796	3.972554	1.244603
24	1	0	0.446699	3.020040	1.747090
25	1	0	0.357822	4.677987	2.046325
26	1	0	-0.510407	3.967519	1.143453
27	1	0	-0.285300	-1.749746	3.859880

Structure 15

1	22	0	-3.631709	-2.338226	-1.176916
2	22	0	-0.339402	-2.084085	-0.552221
3	22	0	-1.113628	1.785836	-0.376039
4	22	0	-4.637544	-0.983827	1.713651
5	22	0	-4.388129	0.972418	-1.124319
6	22	0	-1.370892	-0.390020	2.352488
7	8	0	-1.767884	-2.744902	-1.308529
8	8	0	-4.062780	-0.774306	-1.845129
9	8	0	-4.372755	-2.315963	0.561756
10	8	0	-0.812153	-1.658789	1.056738
11	8	0	-2.668690	1.898024	-1.094843
12	8	0	-1.348827	1.100434	1.204841
13	8	0	-3.172040	-0.777815	2.691386
14	8	0	-4.816850	0.408632	0.692613
15	8	0	-5.593149	1.789502	-1.844256
16	8	0	-0.239334	-0.233075	3.591723
17	6	0	-6.592564	-1.306368	3.126117
18	1	0	-5.757614	-1.207691	3.839469
19	1	0	-7.478639	-1.431073	3.755613
20	1	0	-6.554109	-2.225976	2.518518
21	1	0	-6.801145	-0.392229	2.544590
22	79	0	1.841764	1.708836	0.128083
23	79	0	2.376380	-0.983913	0.026222
24	79	0	0.026981	0.062298	-2.034849
25	79	0	0.368895	3.882922	-0.643482
26	79	0	1.677740	0.179762	2.411068
27	79	0	1.778762	-3.503130	-0.893011

Structure 16

1	22	0	-3.57933621	-2.77709372	-0.92236852
2	22	0	-0.31457621	-2.19156072	-0.38102952
3	22	0	-1.44656221	1.60416228	-0.36826652
4	22	0	-4.59976021	-1.38610872	1.91668648
5	22	0	-4.65227921	0.47211728	-1.04576252
6	22	0	-1.41234321	-0.44706772	2.47197948
7	8	0	-1.69063821	-3.02289572	-1.06449352
8	8	0	-4.15077421	-1.27392372	-1.63261952
9	8	0	-4.29033121	-2.75126672	0.81989548
10	8	0	-0.78679721	-1.72845072	1.21868748
11	8	0	-3.02699421	1.54049828	-1.04516852
12	8	0	-1.57111821	0.97729528	1.24884748
13	8	0	-3.17303221	-0.96278572	2.87098248
14	8	0	-5.10018121	-0.00280672	0.80842148
15	8	0	-5.90049321	1.12059728	-1.85522552
16	8	0	-0.26312021	-0.12404472	3.6600464
17	6	0	-6.59596321	-1.02915972	2.72422748
18	1	0	-6.30744121	-0.89152572	3.77809748

19	1	0	-7.44488921	-0.34637172	2.56313448
20	1	0	-7.00574721	-2.04143772	2.57723148
21	1	0	-6.04080721	-0.18626572	1.56986548
22	79	0	1.50682279	1.82996928	0.02368848
23	79	0	2.28973979	-0.80375072	0.02567548
24	79	0	-0.21525321	-0.09995172	-1.98240952
25	79	0	-0.19444121	3.82484828	-0.76108252
26	79	0	1.56215879	0.40609928	2.37711648
27	79	0	1.91844979	-3.42069672	-0.7178745

Structure 17

1	22	0	3.552466	2.454798	-1.130511
2	22	0	0.264270	2.097996	-0.541874
3	22	0	1.185383	-1.731507	-0.340146
4	22	0	4.578773	1.186562	1.824278
5	22	0	4.459162	-0.813952	-1.090952
6	22	0	1.307975	0.448685	2.390067
7	8	0	1.677031	2.798724	-1.291128
8	8	0	4.091125	0.897751	-1.783125
9	8	0	4.236420	2.436935	0.586428
10	8	0	0.729873	1.691575	1.073435
11	8	0	2.781250	-1.767277	-0.997672
12	8	0	1.349110	-1.054844	1.246318
13	8	0	3.096503	0.834223	2.736342
14	8	0	5.150815	-0.382537	0.772197
15	8	0	5.626241	-1.644435	-1.854904
16	8	0	0.162251	0.255509	3.612987
17	6	0	6.005297	1.822355	3.115721
18	1	0	6.181752	1.051678	3.881960
19	1	0	6.933204	2.033274	2.561477
20	1	0	5.663709	2.742543	3.611729
21	1	0	5.880478	-0.960382	1.084104
22	79	0	-1.775505	-1.772851	0.106019
23	79	0	-2.410225	0.895796	-0.018168
24	79	0	0.016441	-0.067001	-2.029585
25	79	0	-0.192802	-3.889600	-0.628096
26	79	0	-1.707503	-0.230374	2.386426
27	79	0	-1.899424	3.437915	-0.918869

Structure 18

1	22	0	4.220400	1.306643	-0.962322
2	22	0	0.847305	1.823051	-0.442986
3	22	0	0.552344	-2.054554	-0.311790
4	22	0	4.449793	-0.376506	2.052853
5	22	0	4.010996	-2.189483	-0.837804
6	22	0	1.154029	-0.064325	2.521110
7	8	0	2.476652	1.959734	-1.109308
8	8	0	4.420419	-0.445166	-1.813952

9	8	0	4.613528	1.035896	0.694224
10	8	0	1.038243	1.310948	1.181011
11	8	0	2.096063	-2.488015	-0.948414
12	8	0	0.816276	-1.480597	1.296334
13	8	0	2.908513	-0.167850	3.012157
14	8	0	4.477090	-1.763297	0.910683
15	8	0	4.903560	-3.364717	-1.522799
16	8	0	-0.083158	0.083921	3.670662
17	6	0	6.519426	0.373749	3.496475
18	1	0	5.618585	0.601985	4.086610
19	1	0	6.750005	-0.698484	3.501373
20	1	0	7.346908	0.880200	4.003027
21	79	0	-2.326588	-1.189350	0.007602
22	79	0	-2.106895	1.550472	-0.054693
23	79	0	-0.078078	-0.096273	-1.981186
24	79	0	-1.419535	-3.664462	-0.751558
25	79	0	-1.910195	0.218439	2.342195
26	79	0	-0.731238	3.799880	-0.876040
27	1	0	4.819438	-0.553287	-2.703485
28	6	0	5.540464	2.650964	-1.726506
29	1	0	5.459086	3.599441	-1.175341
30	1	0	6.570214	2.275614	-1.631192
31	1	0	5.298899	2.827671	-2.787152
32	1	0	6.502706	0.807701	2.482883

Structure 19

1	22	0	4.086426	1.579763	-1.125344
2	22	0	0.677775	1.664913	-0.684159
3	22	0	0.908143	-2.142162	0.121268
4	22	0	4.488638	0.466723	2.080067
5	22	0	4.366880	-1.873514	-0.498789
6	22	0	1.099862	0.205196	2.546593
7	8	0	2.348042	1.912294	-1.377963
8	8	0	4.600558	-0.206216	-1.683029
9	8	0	4.477193	1.580940	0.632351
10	8	0	0.963238	1.293519	0.973632
11	8	0	2.596948	-2.360445	-0.468464
12	8	0	0.888653	-1.445629	1.791850
13	8	0	2.991503	0.428440	2.936339
14	8	0	4.859677	-1.125678	1.244557
15	8	0	5.356349	-3.017744	-1.095200
16	8	0	-0.108261	0.544440	3.666407
17	6	0	6.311423	-0.046552	3.159976
18	1	0	7.059357	-0.854621	3.184542
19	1	0	6.881760	0.869427	2.930334
20	1	0	5.910736	0.009460	4.183253
21	1	0	5.742270	-0.968537	2.074128
22	79	0	-2.306178	-1.317627	0.244320
23	79	0	-2.221852	1.451981	-0.054780

24	79	0	-0.194235	-0.507901	-1.739252
25	79	0	-1.164004	-3.384531	-1.133707
26	79	0	-2.031815	0.307336	2.414691
27	79	0	-0.962589	3.565525	-1.268499
28	1	0	5.029617	-0.399539	-2.544631
29	6	0	5.151519	3.003501	-2.083811
30	1	0	4.889945	3.991193	-1.676864
31	1	0	6.227215	2.821968	-1.937690
32	1	0	4.900390	2.979630	-3.155878

Structure 20

1	22	0	3.891994	1.677655	-0.808334
2	22	0	0.552684	1.810724	-0.547406
3	22	0	1.261469	-2.059503	0.243845
4	22	0	4.130248	0.694948	2.111565
5	22	0	4.615497	-1.475015	-0.144982
6	22	0	0.930383	0.360162	2.655765
7	8	0	2.252218	2.223054	-1.168304
8	8	0	4.770164	0.091103	-1.502955
9	8	0	3.395656	0.421775	0.545261
10	8	0	0.690411	1.533069	1.141465
11	8	0	2.990920	-2.338819	-0.187328
12	8	0	1.012064	-1.282889	1.861236
13	8	0	2.769113	0.781930	3.183191
14	8	0	5.206639	-0.887316	1.757508
15	8	0	5.822633	-2.466378	-0.605524
16	8	0	-0.376838	0.454234	3.705461
17	6	0	4.981844	2.540472	1.483752
18	1	0	5.045811	2.837883	2.555088
19	1	0	6.013047	2.428096	1.122479
20	1	0	4.541817	3.434930	1.021152
21	1	0	5.856067	-1.423467	2.252306
22	79	0	-2.108647	-1.576460	0.136652
23	79	0	-2.339767	1.187402	-0.152537
24	79	0	0.065885	-0.467004	-1.615858
25	79	0	-0.603611	-3.415193	-1.222437
26	79	0	-2.204776	0.044917	2.326623
27	79	0	-1.284489	3.437224	-1.322576
28	1	0	5.311577	-0.032974	-2.308022
29	6	0	5.078193	3.184572	-1.534127
30	1	0	4.457811	4.088681	-1.629641
31	1	0	6.032881	3.435605	-1.066476
32	1	0	5.263808	2.785584	-2.554181

Structure 21

1	22	0	3.888749	1.619575	-0.709450
2	22	0	0.566575	1.817830	-0.531535
3	22	0	1.254781	-2.064353	0.234908

4	22	0	4.114809	0.684083	2.140315
5	22	0	4.608793	-1.498857	-0.131380
6	22	0	0.910613	0.334797	2.657692
7	8	0	2.263786	2.261903	-1.094507
8	8	0	4.820301	0.078431	-1.463494
9	8	0	3.341925	0.364893	0.542557
10	8	0	0.692396	1.524629	1.160079
11	8	0	2.983703	-2.358887	-0.194902
12	8	0	0.985540	-1.302677	1.857089
13	8	0	2.763714	0.708136	3.206134
14	8	0	5.173651	-0.901926	1.780547
15	8	0	5.818078	-2.489493	-0.589711
16	8	0	-0.394138	0.435820	3.709131
17	6	0	5.038253	2.648700	0.721669
18	1	0	5.005789	2.135389	2.293870
19	1	0	6.121079	2.510300	0.786859
20	1	0	4.734218	3.626109	1.105966
21	1	0	5.816035	-1.430884	2.292931
22	79	0	-2.113714	-1.569054	0.124114
23	79	0	-2.333386	1.196039	-0.153599
24	79	0	0.069631	-0.459104	-1.617517
25	79	0	-0.612822	-3.407384	-1.237694
26	79	0	-2.211012	0.043441	2.319435
27	79	0	-1.269392	3.446976	-1.311816
28	1	0	5.356316	-0.062894	-2.268374
29	6	0	5.150708	3.294020	-1.253731
30	1	0	4.305299	3.842001	-1.693936
31	1	0	5.869820	4.019715	-0.874454
32	1	0	5.675858	2.667461	-1.996927

Structure 22

1	22	0	3.849819	1.573652	-0.753334
2	22	0	0.564676	1.832608	-0.502398
3	22	0	1.236540	-2.078981	0.203798
4	22	0	4.125166	0.522294	2.342036
5	22	0	4.628410	-1.502689	-0.139104
6	22	0	0.887045	0.284774	2.667364
7	8	0	2.240424	2.290276	-1.097004
8	8	0	4.838198	0.055233	-1.484810
9	8	0	3.473762	0.517884	0.635259
10	8	0	0.708891	1.496204	1.182757
11	8	0	2.977921	-2.306274	-0.220329
12	8	0	0.974122	-1.338814	1.831748
13	8	0	2.709138	0.558268	3.308856
14	8	0	5.152759	-1.036577	1.809350
15	8	0	5.803274	-2.525359	-0.608674
16	8	0	-0.423156	0.372131	3.713330
17	6	0	5.058390	2.873891	0.223348
18	1	0	5.047354	1.798227	2.835429

19	1	0	5.951282	2.443933	0.688085
20	1	0	4.573447	3.565486	0.917339
21	1	0	5.794791	-1.610438	2.272803
22	79	0	-2.122448	-1.562528	0.093336
23	79	0	-2.330884	1.207649	-0.140999
24	79	0	0.072711	-0.433496	-1.623671
25	79	0	-0.624303	-3.386717	-1.294349
26	79	0	-2.219533	0.014765	2.315179
27	79	0	-1.254031	3.474486	-1.259646
28	1	0	5.361427	-0.110391	-2.293116
29	6	0	5.368854	3.523047	-1.152234
30	1	0	4.500531	4.022555	-1.606722
31	1	0	6.128530	4.305515	-1.024584
32	1	0	5.820708	2.824700	-1.881892

Structure 23

1	22	0	-3.674529	-2.519061	-1.031681
2	22	0	-0.351626	-1.867084	-0.684210
3	22	0	-1.625208	1.671342	0.210502
4	22	0	-4.286354	-1.625884	2.312667
5	22	0	-4.963990	0.491711	-0.268371
6	22	0	-1.030277	-0.615019	2.593988
7	8	0	-1.897302	-2.535534	-1.371020
8	8	0	-4.828489	-1.024481	-1.607896
9	8	0	-4.086885	-2.547590	0.710460
10	8	0	-0.677913	-1.618064	0.991381
11	8	0	-3.348259	1.344085	-0.240692
12	8	0	-1.287696	1.050327	1.864223
13	8	0	-2.772343	-1.249447	3.093904
14	8	0	-5.389594	-0.128235	1.607122
15	8	0	-6.158824	1.484391	-0.748839
16	8	0	0.267114	-0.630041	3.666577
17	1	0	-6.085090	0.371975	2.082961
18	79	0	1.705877	1.814941	0.224851
19	79	0	2.395577	-0.856402	-0.146149
20	79	0	-0.154445	0.478381	-1.722635
21	79	0	-0.048121	3.494011	-1.049044
22	79	0	2.004407	0.144931	2.363032
23	79	0	1.733889	-3.233056	-1.344408
24	1	0	-5.307256	-0.909052	-2.454422
